# Maggot Secretions Skew Monocyte-Macrophage Differentiation Away from a Pro-Inflammatory to a Pro-Angiogenic Type

**DOI:** 10.1371/journal.pone.0008071

**Published:** 2009-11-30

**Authors:** Mariena J. A. van der Plas, Jaap T. van Dissel, Peter H. Nibbering

**Affiliations:** 1 Department of Infectious Diseases, Leiden University Medical Center, Leiden, The Netherlands; 2 Department of Surgery, Leiden University Medical Center, Leiden, The Netherlands; Charité-Universitätsmedizin Berlin, Germany

## Abstract

**Background:**

Maggots of the blowfly *Lucilia sericata* are used for the treatment of chronic wounds. Earlier we reported maggot secretions to inhibit pro-inflammatory responses of human monocytes. The aim of this study was to investigate the effect of maggot secretions on the differentiation of monocytes into pro-inflammatory (MØ-1) and anti-inflammatory/pro-angiogenic macrophages (MØ-2) as these cells play a central role in wound healing.

**Methodology/Principal Findings:**

Freshly isolated monocytes were incubated with secretions and GM-CSF or M-CSF for 6 days and then stimulated with LPS or LTA for 18 h. The expression of cell surface molecules and the levels of cytokines, chemokines and growth factors in supernatants were measured. Our results showed secretions to affect monocyte-macrophage differentiation leading to MØ-1 with a partial MØ-2-like morphology but lacking CD163, which is characteristic for MØ-2. In response to LPS or LTA, secretions-differentiated MØ-1 produced less pro-inflammatory cytokines (TNF-α, IL-12p40 and MIF) than control cells. Similar results were observed for MØ-2 when stimulated with low concentrations of LPS. Furthermore, secretions dose-dependently led to MØ-1 and MØ-2 characterized by an altered chemokine production. Secretions led to MØ-2, but not MØ-1, producing enhanced levels of the growth factors bFGF and VEGF, as compared to control cells. The expression of cell-surface receptors involved in LPS/LTA was enhanced by secretions, that of CD86 and HLA-DR down-regulated, while receptors involved in phagocytosis remained largely unaffected.

**Conclusions:**

Maggot secretions skew the differentiation of monocytes into macrophages away from a pro-inflammatory to a pro-angiogenic type.

## Introduction

Foot ulcers of patients with diabetes mellitus are associated with tremendous health care related and social costs [1], [2]. It has been observed that only two-thirds of foot ulcers will heal [3]–[5]. Healing of foot ulcers is essential, since a relatively high proportion will result in amputation, leading to further costs and patient suffering [6], [7]. Sterile larvae -maggots- of the blowfly *Lucilia sericata* are used for the treatment of different types of wounds including diabetic foot ulcers [8]–[11]. Clinical observations indicate that besides the removal of necrotized tissue and infectious microorganisms, maggots actively promote healing of chronic wounds [8], [12], [13]. Earlier we reported that maggot secretions inhibited the pro-inflammatory responses of human neutrophils [14] and monocytes [15] through elevation of cyclic AMP. In response to local factors, monocytes migrate into the inflamed site where they may differentiate into macrophages which exhibit either pro-inflammatory or anti-inflammatory/pro-angiogenic functions. These divergent functions of macrophages are dependent mainly on the macrophage subset which is regulated by cytokines and growth factors present in the local micro-environment [16]. For example, monocytes incubated in the presence of granulocyte macrophage-colony stimulating factor (GM-CSF) develop in pro-inflammatory macrophages (MØ-1), i.e. fried egg-shaped macrophages displaying high IL-12 and low IL-10 production in response to lipopolysaccharides (LPS), while monocytes incubated with macrophage-colony stimulating factor (M-CSF) differentiate to anti-inflammatory/pro-angiogenic macrophages (MØ-2), characterized by a stretched, spindle-like morphology, expression of CD163, and low IL-12 and high IL-10 production in response to LPS. Pro-inflammatory macrophages, by secreting cytokines and chemokines, are responsible for recruiting and activating immune cells such as neutrophils, monocytes and macrophages involved in elimination of infectious agents [17]. In addition, these cytokines lead to the expression of co-stimulatory molecules on macrophages essential for T-cell activation. When the infection is cleared, the balance shifts form pro-inflammatory macrophages to macrophages with anti-inflammatory/pro-angiogenic cytokine and growth factor activities. These cells are involved in clearance of apoptotic cells [18], [19], neovascularisation and fibroblast and epidermal cell proliferation [20]. Concurrently, these cells play a major role in matrix synthesis by secretion of basement membrane components, such as collagen [21], [22].

Diabetic foot wounds are marked by a prolonged and dysregulated inflammatory phase. The balance between pro-inflammatory and anti-inflammatory macrophages is disturbed [23] resulting in an enhanced production and release of pro-inflammatory cytokines, proteases and reactive oxygen species which lead to growth factor inactivation and tissue destruction [24], [25]. Therefore, inhibition of pro-inflammatory responses of these cells may restrict their deleterious effects, whereas the induction of anti-inflammatory/pro-angiogenic cytokine and growth factor activities may contribute to wound repair. Based on the above considerations, the aim of this study was to investigate the effects of maggot secretions on the differentiation of monocytes into pro-inflammatory and anti-inflammatory/pro-angiogenic macrophages. Our findings provide novel insights into the modes of action of maggot therapy.

## Materials and Methods

### Maggots and Maggot Secretions

Sterile second- and third-instar larvae of *L. sericata* were a kind gift from BioMonde GmbH (Barsbüttel, Germany). Maggot secretions were collected as described previously [15]. Prior to use, sterile preparations of secretions were pooled and centrifuged at 1,300×g for 5 min at 4°C to remove particulate material. Subsequently, protein concentrations of the pools were determined using the Pierce BCA Protein Assay kit (Pierce Biotechnology, Rockford, IL, USA) according to manufacturer's instructions.

### Isolation of Human Monocytes

Peripheral blood mononuclear cells from healthy donors were isolated from buffy coats by Ficoll Amidotrizoate (*ρ* = 1.077 g/ml) density centrifugation at 700×g for 20 min. Cells from the interphase were washed three times with PBS (pH 7.4) and monocytes were purified using anti-CD14-coated Microbeads (Miltenyi Biotec GmbH, Bergisch Gladbach, Germany) according to manufacturer's instructions. Next, cells were centrifuged and resuspended in RPMI 1640 supplemented with 2 mmol/l glutamax-I/glutamine, 2 mmol/l penicillin/streptomycin and 10% (vol./vol.) inactivated fetal calf serum (further referred to as standard medium).

### Macrophages

MØ-1 and MØ-2 were obtained by culturing 3×10^5^ monocytes/ml of standard medium in 24-wells plates in the presence of respectively 5 ng of recombinant GM-CSF/ml (Biosource, Camarillo, Ca, USA) or 12.5 ng of recombinant M-CSF/ml (R&D Systems Europe Ltd., Abingdon, UK). After 6 days, macrophages were stimulated with LPS (0.01–100 ng/ml; Sigma Chemical Co., St. Louis, MO, USA), lipoteichoic acid (LTA; 0.01–5 µg/ml; Invivogen, Toulouse, France) or no stimulus (further referred to as naïve macrophages). After 18 to 20 h incubation at 37°C and 5% CO_2_, supernatants were collected and stored at −70°C. To investigate the effect of secretions on the differentiation of monocytes to macrophages, secretions (range 0.35–70 µg/ml) were added to the wells at the start of the culture and the resulting macrophages are further referred to as secretions-differentiated macrophages.

### Measurement of the Levels of Cytokines, Chemokines and Growth Factors

The levels in the supernatants of the cell cultures were assessed using BioSource CytoSet™ (Biosource Europe, S.A., Belgium) and Bio-Plex kits (BIO-RAD, Hercules, CA, USA) according to manufacturer's instructions.

### Flow Cytometry

To verify the differentiation of monocytes into MØ-1 and MØ-2, macrophages were incubated with phycoerythrin-conjugated monoclonal antibodies (mAbs) directed against CD163 purchased from BD Pharmingen™ (BD BioSciences, Erembodegem, Belgium). Furthermore, cells were incubated with FITC- or phycoerythrin-conjugated mAbs directed against CD11b, CD14, CD32, CD35, CD54, CD64, CD86, HLA-DR and CD206 (BD Pharmingen™, BD BioSciences, Erembodegem, Belgium), CD16 (EuroBioSciences GmbH, Friesoythe, Germany), and CD282 (Toll-like receptor [TLR]-2) and CD284 (TLR-4; Hycult Biotechnology, Uden, the Netherlands for both); incubation was in PBS containing 0.5% (wt/vol.) BSA for 30 min on ice. Analyses were performed on the FACSCalibur (Becton&Dickinson, La Jolla, CA, USA) in combination with CellQuest™ Pro 4.0.2 software. Mean fluorescence intensities (MFI) of unstained samples were subtracted from the stained samples. MFI's below 6 are indicated as not detectable (≤2 times MFI unstained samples).

### Statistical Analysis

Differences between the values for cells incubated in the presence of maggot secretions and those for cells incubated with H_2_O were analysed using a Wilcoxon test using Graphpad Prism version 4.02.

## Results

### Effect of Maggot Secretions on the Differentiation of Monocytes to Macrophages

**Figure 1:**
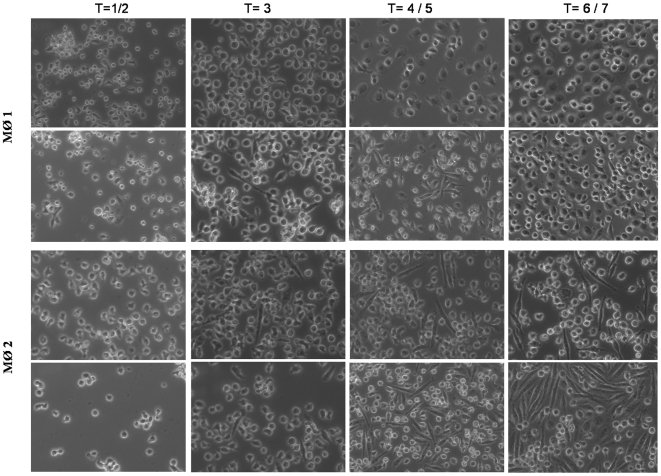
Light microscopy analysis of the effect secretions on the differentiation of monocytes to macrophages. Monocytes were differentiated to MØ-1 in the presence of GM-CSF (A) or in the presence of GM-CSF and 35 µg of secretions/ml (B) and their morphology evaluated. Similarly, monocytes were differentiated to MØ-2 in the presence of M-CSF (C) and in the presence of M-CSF and secretions (D). Results indicated in days are from a representative experiment.

Light microscopy revealed macrophages that differentiated under the influence of GM-CSF to display a ‘fried egg-like’ morphology (Fig. 1A) whereas the addition of secretions (35 µg/ml) led to MØ-1 (Fig. 1B) that partially obtained a phenotype resembling MØ-2, i.e. elongated, spindle-like appearance as induced by M-CSF (Fig. 1C). Secretions enhanced the development of this morphology by M-CSF differentiated macrophages (Fig. 1D). However, secretions did not induce CD163 expression on GM-CSF-differentiated macrophages, which is a characteristic of MØ-2. In addition, secretions did not lead to an altered expression of CD163 (mean fluorescence intensity ∼20) on MØ-2.

**Figure 2:**
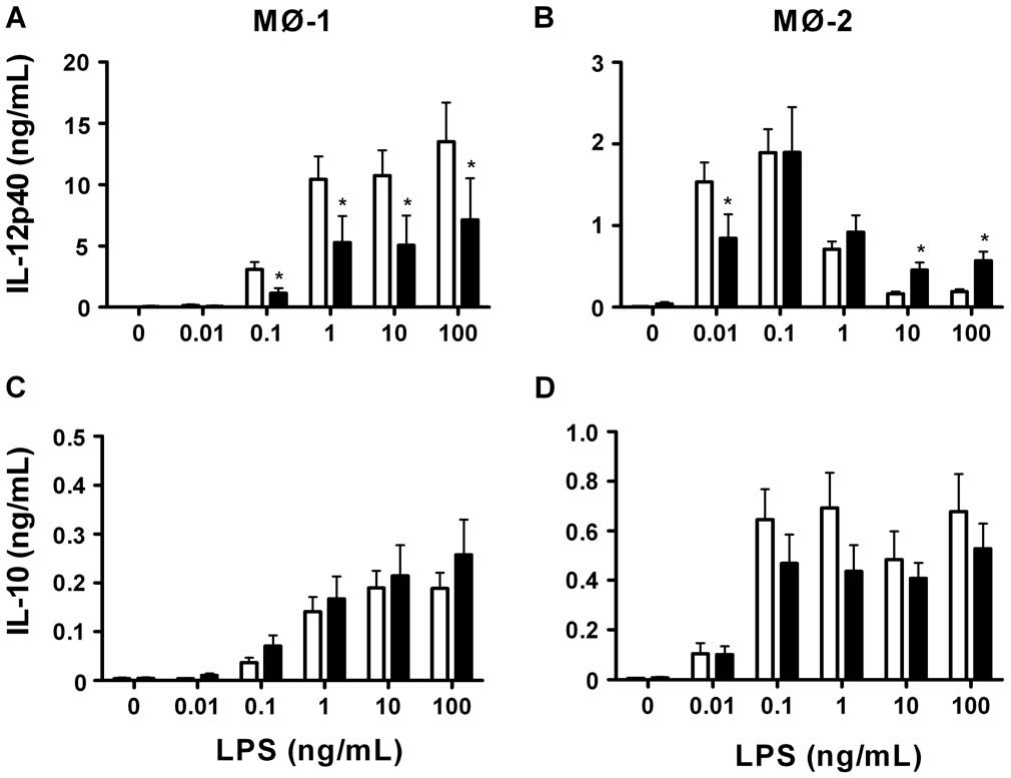
Cytokine production in response to LPS. We measured the production of IL-12p40 (A,B) and IL-10 (C,D) by control and secretions-differentiated MØ-1 and MØ-2 in response to a range of LPS. The results, expressed in ng/ml, are means±SEM of 9–10 experiments. Open bars: control macrophages; filled bars: secretions differentiated macrophages. *p<0.05 for differences from control macrophages.

Further investigations showed secretions to affect macrophage differentiation resulting in MØ-1 that in response to various concentrations of LPS produced less IL-12p40 than control macrophages (Fig. 2A). MØ-2 differentiated in the presence of secretions produced less IL-12p40 upon stimulation with 0.01 ng of LPS/ml, whereas 10 and 100 ng/ml led to increased IL-12p40 production compared to control MØ-2 (Fig. 2B). Remarkably, the production of IL-12p40 by MØ-2 was almost 10 times higher in response to 0.01 ng of LPS/ml as compared to 100 ng of LPS/ml. The production of IL-10 by both types of macrophages differentiated in the presence of secretions did not differ from that by control macrophages (Fig. 2C and 2D). Taken together, the above results indicate that maggot secretions affect the differentiation of monocytes to macrophages, but do not result in the differentiation from one type into the other.

### Cytokine Production by Secretions Differentiated Macrophages

The results showed that maggot secretions dose-dependently gave rise to MØ-1 with a decreased production of the pro-inflammatory cytokines IL-12p40, TNF-α, and Macrophage Migration Inhibitory Factor (MIF) upon LPS stimulation, as compared to control cells (Table 1), whereas the level of IL-1β (mean 103 and range 51–150 pg/ml) did not differ (data not shown). In addition, the LPS-induced production of IL-6 by these cells was dose-dependently enhanced as was that of the anti-inflammatory cytokine IL-10 when using small amounts of secretions. Secretions had no effect on base-line levels of IL-12p40, TNF-α, IL-10 and IL-1β (data not shown), but 70 µg of secretions/ml led to cells with an increased (p<0.005) production of IL-6 from 37 (range: 0–125) to 128 (range: 6–524) pg/ml and a decreased (p<0.05) production of MIF from 226 (range: 81–553) to 65 (range: 0–99) pg/ml.

**Table 1:** LPS-induced cytokine production by secretions differentiated macrophages.

	Control cells				Macrophages differentiated in the presence of secretions (µg/mL)			
	Type MØ	LPS (ng/mL)	Median (ng/mL)	Range (ng/mL)	0.35	3.5	35	70
					(%)	(%)	(%)	(%)
IL-12p40	1	100	25	7.6–38.2	74±6**	75±5**	68±9*	35±6**
	2	0.01	1	0.2–3.7	72±4**	70±6**	33±6**	22±3**
	2	100	0.2	0.07–0.5	77±4**	71±7**	176±22**	160±28*
TNF-α	1	100	51	25.2–131	92±5	85±5*	71±6**	53±9**
	2	0.01	2.8	0.9–7.2	90±4*	95±7	58±6**	30±3**
	2	100	3.8	2.6–5.8	97±5	100±6	97±8	63±9*
MIF	1	100	0.2	0.07–0.4	60±10*	42±7**	19±5**	24±7**
	2	0.01	0.8	0.2–1.1	70±13	49±9*	19±3*	15±1*
	2	100	0.3	0.08–0.7	87±23	35±10**	19±5**	6±3**
IL-6	1	100	37	3.4–60.1	108±8	135±12**	193±42**	226±52*
	2	0.01	1.9	1.5–27.4	122±9*	162±14**	239±28**	208±26**
	2	100	0.7	0.2–1.4	106±8	148±14**	427±62**	690±89**
IL-10	1	100	0.1	0–0.6	132±14*	137±16*	98±16	97±15
	2	0.01	0.04	0.02–1.3	93±8	114±13	74±12	83±14
	2	100	0.7	0.2–1.0	102±5	101±8	87±8	67±9*

The results for the control macrophages are expressed as the median value and range, and are set at 100%. The effect of secretions is expressed as a percentage relative to the cytokine production by control cells. Results are means±SEM of at least six experiments. Values are significantly (*p<0.05 and **p<0.005) different from those for macrophages stimulated with LPS.

In agreement with their effects on LPS-stimulated MØ-1, maggot secretions dose-dependently led to MØ-2 that showed a reduced production of IL-12p40 and TNF-α when stimulated with 0.01 ng of LPS/ml, as compared to control cells (Table 1). The levels of these cytokines were dose-dependently altered in the presence of secretions when stimulated with 100 ng of LPS/ml. Furthermore, secretions differentiated MØ-2 showed a reduced MIF production and an increased IL-6 production regardless of the amount of LPS used. IL-10 production was not altered by these cells when stimulated with 0.01 ng of LPS/ml, whereas 70 µg of secretions/ml led to cells with decreased IL-10 levels when stimulated with 100 ng of LPS/ml. IL-1β levels were not detectable in the supernatants of MØ-2. Secretions had no effect on base-line levels of TNF-α, IL-12p40 and IL-10 (data not shown), but 70 µg of secretions/ml led to MØ-2 displaying increased (p<0.05) production of IL-6 from 8 (range: 0–42) to 31 (range: 0–78) pg/ml and decreased (p<0.005) production of MIF from 291 (range: 165–567) to 33 (range: 0–106) pg/ml.

### Chemokine Production by Secretions Differentiated Macrophages

As influx of inflammatory cells contributes to excessive inflammation in chronic wounds, we investigated the chemokine profile of secretions-differentiated and control macrophages. The results (Table 2) showed that maggot secretions dose-dependently gave rise to naïve and LPS-stimulated MØ-1 and MØ-2 displaying increased production of Monocyte Chemotactic Protein-1 (MCP-1) and IL-8, but decreased production of Macrophage Inflammatory Protein-1β (MIP-1β). Furthermore, the production of RANTES was reduced by these cells when stimulated with LPS. RANTES was not detectable (<10 pg/ml) in the supernatants of naïve cells, but 70 µg of secretions/ml led to MØ-1 producing 20 (range: 4.5–661) pg/ml and to MØ-2 producing 13 (range: 3–24) pg/ml of this chemokine.

**Table 2:** LPS-induced chemokine production by secretions differentiated macrophages.

	Control cells				Macrophages differentiated in the presence of secretions (µg/mL)			
	Type MØ	LPS (ng/mL)	Median (ng/mL)	Range (ng/mL)	0.35 (%)	3.5 (%)	35 (%)	70 (%)
IL-8	1	0	1.0	0.4–6.6	146±22	467±98*	2761±923*	2906±996*
	1	100	59	21–116	118±14	176±28*	343±72*	338±83*
	2	0	1.5	0.4–2.2	103±15	142±10*	552±158*	716±129*
	2	0.01	12	6.3–24	110±19	149±30*	250±34*	378±66*
	2	100	4.5	3.8–7.6	117±10	173±22*	480±67*	703±112*
MCP-1	1	0	0.7	0.2–5.8	166±25*	437±160*	990±353*	1127±471*
	1	100	8.7	3.9–49	135±22	208±50*	335±256*	368±85*
	2	0	31	5.9–40	137±8*	159±12*	318±83*	361±106*
	2	0.01	63	32–122	111±9	161±15*	189±17*	175±10*
	2	100	53	9.0–61	134±35	157±23*	272±48**	242±42**
MIP-1β	1	0	0.09	0.02–0.5	71±5*	42±7**	16±4**	14±4*
	1	100	25	1.2–338	118±37	68±15	28±12*	6±2**
	2	0	0.09	0.03–1.3	93±4	55±8**	22±6**	17±5**
	2	0.01	48	24–84	87±10	51±6**	6±1**	4±1**
	2	100	45	25–75	92±6	61±4**	9±2**	4±1**
RANTES	1	0	ND					
	1	100	4.6	2.1–15	129±30	79±19	28±18**	21±6**
	2	0	ND					
	2	0.01	0.5	0.2–1.5	48±11**	62±8**	46±8**	50±8*
	2	100	2.0	1.2–3.4	90±4	78±8*	47±4**	34±4**

The results for control macrophages are expressed as the median value and range, and are set at 100%. The effect of secretions is expressed as a percentage relative to the chemokine production by control cells. Results are means±SEM of at least six experiments. Values are significantly (*p<0.05 and **p<0.005) different from those for macrophages stimulated with LPS. ND: not detectable.

### Growth Factor Production by Secretions Differentiated Macrophages

Since tissue synthesis and neovascularisation are essential for wound healing, we investigated the production of growth factors by macrophages differentiated in the presence of secretions. The results showed similar levels of basic fibroblast growth factor (bFGF) and vascular endothelial growth factor (VEGF) in the supernatants of secretions-differentiated MØ-1 and control cells; VEGF production by naïve cells was not detectable (Table 3). Furthermore, secretions led to MØ-1 showing decreased production of platelet derived growth factor (PDGF)-BB upon LPS stimulation, whereas the production of G-CSF (median: 306 (46–1060) pg/ml) by these cells did not differ (data not shown); PDGF-BB and G-CSF were not detectable in supernatants from naïve MØ-1. Remarkably, secretions (70 µg/ml) dose-dependently led to naïve MØ-1 displaying (p<0.005) reduced levels of GM-CSF from 665 (range: 22–2505) to 138 (range: 1–1624) pg/ml and when stimulated with LPS from 556 (range: 62–1969) to 110 (range: 0–1216) pg/ml.

**Table 3:** LPS-induced growth factor production by secretions differentiated macrophages.

	Control cells				Macrophages differentiated in the presence of secretions (µg/mL)			
	Type MØ	LPS (ng/mL)	Median (pg/mL)	Range (pg/mL)	0.35 (%)	3.5 (%)	35 (%)	70 (%)
bFGF	1	0	51	0–111	98±6	92±12	116±25	150±36
	1	100	58	22–117	97±3	98±4	95±7	114±8
	2	0	34	10–108	170±37	199±48*	305±86*	372±114*
	2	0.01	27	14–40	74±19	180±28*	234±44*	288±56**
	2	100	56	32–107	109±4*	130±13*	180±30*	214±42*
VEGF	1	0	ND					
	1	100	25	0–120	87±5	94±8	101±16	95±20
	2	0	99	18–195	172±41*	254±45**	545±106**	627±108**
	2	0.01	90	21–199	126±35	825±232*	1168±403*	1382±579**
	2	100	342	225–540	129±10*	174±18**	415±57**	423±103**
PDGF	1	0	ND					
	1	100	15	8–61	105±6	98±9	72±5*	64±9*
	2	0	20	6–95	115±13	102±8	62±10*	58±16*
	2	0.01	49	24–105	63±11*	117±14	54±7**	40±4**
	2	100	25	13–114	123±6	112±7	67±3*	57±5*

The results for control macrophages are expressed as the median value and range, and are set at 100%. The effect of secretions is expressed as a percentage relative to the production of growth factors by control cells. Results are means±SEM of at least six experiments. Values are significantly (*p<0.05 and **p<0.005) different from those for macrophages stimulated with LPS. ND: not detectable.

Contrastingly to MØ-1, secretions dose-dependently gave rise to naïve and LPS-stimulated MØ-2 with increased production of bFGF and VEGF. Additionally, 100 ng of LPS/ml induced a higher production of VEGF by MØ-2 than 0.01 ng of LPS/ml, while the effect of secretions was higher when these cells when stimulated with the latter amount of LPS. Furthermore, secretions gave rise to MØ-2 showing decreased production of PDGF-BB in response to LPS; no PDGF-BB was detected in the supernatants of naïve MØ-2. G-CSF and GM-CSF were not detectable in the supernatants of MØ-2 (data not shown).

### Expression of Cell Surface Receptors on Secretions Differentiated Macrophages

To further investigate the effect of secretions on the differentiation of monocytes into macrophages, we measured the expression of cell surface molecules involved in pathogen recognition, opsono-phagocytosis, cell adhesion and T-cell activation.

The results showed that secretions (35 µg/ml) gave rise to naïve and LPS-stimulated MØ-1 and MØ-2 displaying increased expression of the pathogen-recognition receptors TLR-2 and TLR-4, as compared to control cells (Table 4). Interestingly, LPS down-regulated the expression of these two receptors on MØ-1 (p<0.005) but up-regulated their expression on MØ-2 (p<0.05). Additionally, CD14 was completely down-regulated during the differentiation of monocytes to MØ-1 but remained present on these cells when differentiated in the presence of secretions. The expression of CD14 on MØ-2 was not affected. The expression of the mannose receptor CD206 was increased on secretions differentiated MØ-1, but not on MØ-2.

**Table 4:** Expression of surface receptors by secretions-differentiated macrophages.

		MØ-1				MØ-2					
		No stimulus		LPS (100 ng/mL)		No stimulus		LPS (0.01 ng/mL)		LPS (100 ng/mL)	
		0	35 µg/mL	0	35 µg/mL	0	35 µg/mL	0	35 µg/mL	0	35 µg/mL
CD282	TLR-2	77±6	103±12*	40±4	62±7*	54±4	65±6*	65±6	78±8*	72±7	86±7*
CD284	TLR-4	50±4	68±10*	32±3	45±5*	43±3	51±4*	55±5	66±6**	58±5	68±5**
CD14	LPS-R	ND	8±2*	ND	11±2*	42±4	39±2	41±1	41±3	24±2	30±3
CD206	MMR	45±6	57±7*	22±2	30±3*	32±4	36±3	27±4	34±5	24±3	27±4
CD64	FCγRI	22±3	23±3	12±2	12±2	10±1	13±2	11±2	13±3	15±3	18±3
CD32	FCγRII	22±5	19±3	19±2	24±4	184±19	162±21*	206±23	195±26	170±18	181±18
CD16	FCγRIIIA	9±1	17±4*	6±0.4	12±2*	12±3	25±8*	25±4	37±6*	11±3	23±9*
CD35	CR1	23±3	30±4*	8±1	9±1	15±1	15±1	12±1	13±1	8±1	8±1
CD11b	CR3	113±23	171±40*	80±8	112±19*	231±12	235±16	191±14	207±15	188±17	206±18
CD54	ICAM-1	156±17	192±27	374±60	355±66	365±27	385±19	920±115	911±134	934±107	885±105
CD86	B7.2	19±3	14±2*	105±15	39±5*	77±7	61±6**	35±2	29±2*	69±6	42±3**
HLA-DR		212±36	218±34	270±40	249±41	317±42	250±25*	233±32	201±21*	313±43	239±21*

Results, expressed as the mean fluorescence intensity (MFI), are means±SEM of 6–11 experiments. Values are significantly (*p<0.05 and **p<0.005) different from those for control cells. ND: not detectable.

Furthermore, the expression of Fcγ-receptor III, but not Fcγ-receptor II, was increased on naïve and LPS-stimulated MØ-1 and MØ-2 differentiated in the presence of secretions. Fcγ-receptor II was slightly decreased on naïve MØ-2. Secretions led to naïve MØ-1 showing enhanced expression of complement receptor 1 but no effect was seen on MØ-2 and LPS-stimulated MØ-1. In addition, the expression of CD11b (complement receptor 3, together with CD18) was enhanced by secretions differentiated MØ-1 but not affected on MØ-2. The expression of cell adhesion receptor ICAM-1 was not affected by secretions. Finally, secretions led to MØ-1 and MØ-2 with reduced expression of the co-stimulatory molecule B7.2, and to MØ-2, but not MØ-1, with decreased expression of HLA-DR.

### Cytokine Production by Secretions Differentiated Macrophages in Response to LTA

**Figure 3:**
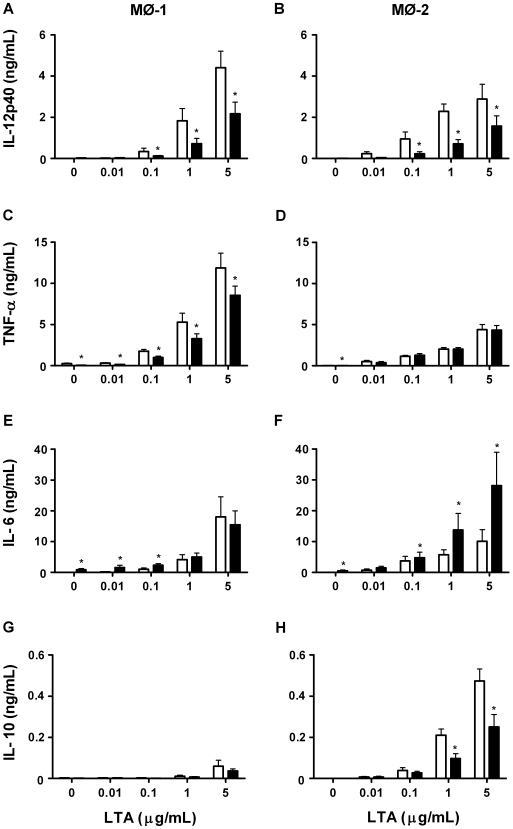
Cytokine production in response to LTA. We measured the production of IL-12p40 (A,B), TNF-α (C,D), IL-6 (E,F) and IL-10 (G,H) by control and secretions-differentiated MØ-1 and MØ-2 induced by a range of LTA. The results, expressed in ng/ml, are means±SEM of 12 experiments. Open bars: control macrophages; filled bars: secretions differentiated macrophages. *p<0.05 for differences from control macrophages.

The results showed that secretions gave rise to MØ-1 (Fig. 3A) and MØ-2 (Fig. 3B) displaying reduced levels of IL-12p40, as compared to control cells, from 0.1 ng of LTA/ml. Furthermore, secretions differentiated MØ-1 (Fig. 3C), but not MØ-2 (Fig. 3D), showed reduced TNF-α production regardless of the amount of LTA used. In addition, the production of IL-6 was enhanced by naïve MØ-1 (Fig. 3E) and by MØ-2 for all conditions (Fig. 3F) when differentiated in the presence of secretions. Secretions had no effect on the production of IL-10 by MØ-1 (Fig. 3G) but led to MØ-2 showing reduced levels of this cytokine when using 1–5 ng of LTA/ml (Fig. 3H).

## Discussion

The main conclusion from the present study is that maggot secretions skew the monocyte-macrophage differentiation away from a pro-inflammatory to a pro-angiogenic type. This conclusion is based on the following observations. First, maggot secretions dose-dependently led to MØ-1 producing less IL-12p40, TNF-α and MIF upon LPS stimulation as compared to control cells. Similar results were obtained for MØ-2 upon stimulation with low amounts of LPS. These actions of maggot secretions on the differentiation of macrophages are not limited to stimulation via TLR-4 as similar effects were observed when stimulated via the TLR-2 pathway. Interestingly, adding secretions (35 µg/ml) to fully differentiated macrophages did not lead to a reduced production of IL-12p40 or TNF-α (data not shown), indicating that secretions effect the differentiation of the cells. Second, maggot secretions led to MØ-1 and MØ-2 with a reduced production of the chemokines MIP-1β, RANTES and PDGF-BB and an increased production of MCP-1 and IL-8. Based upon these findings, it is not possible to predict the overall effect of maggot secretions on migration of leukocytes into the inflamed site. However, earlier we reported that secretions reduce the migration of both monocytes [15] and neutrophils [14] irrespective of the presence of chemokines. Therefore, migration of leukocytes will likely be reduced in the presence of secretions. Third, secretions dose-dependently led to MØ-2, but not MØ-1, with enhanced production of bFGF and VEGF. These growth factors, together with IL-8, are involved in endothelial cell migration and proliferation which is essential for angiogenesis [20], [26]. In addition, low amounts of TNF-α, as observed after exposure of secretions-differentiated macrophages to LPS, are known to induce angiogenesis as well. The exact roles of the elevated IL-6 production by secretions-differentiated macrophages in inflammation and neovascularisation are unclear as this cytokine often exerts its effects by regulating the production of other molecules, such as MIP-1 [27], which we did not observe.

Other findings pertain to the effect of maggot secretions on monocyte-macrophage differentiation with regard to the expression of cell-surface receptors. First, secretions led to MØ-1 and MØ-2 with increased expression of TLR2 and TLR4, as compared to control cells. Additionally, the expression of the mannose receptor CD206 was increased by secretions-differentiated MØ-1 while the CD14 expression was still detectable on these cells. These results suggest that secretions-differentiated macrophages may become more sensitive to pathogen-associated molecular patterns, like LPS and LTA. However, we found no enhanced sensitivity of the cells to these stimuli. Consequently, our results may be caused by interference of secretions with signal transduction pathways down stream of receptor activation such as a transient rise in cAMP [28], [29] and is reported for monocytes[15] and neutrophils [14] after exposure to secretions. Second, secretions differentiated MØ-1 and MØ-2 displayed enhanced expression of CD16. Additionally, the expression of CD11b (part of CR3), involved in both phagocytosis and adhesion to endothelial cells [30], was enhanced on secretions differentiated MØ-1. Expression of CD35 (CR1) was enhanced only on naïve MØ-1. Together, it will be of interest to investigate whether the increased expression of above mentioned receptors mediate phagocytosis of pathogens by macrophages. Third, secretions led to MØ-1 with decreased expression of the co-stimulatory molecule B7.2. This may indicate a reduction in MØ-1-induced Th1 cell proliferation and function [31], [32]. As MØ-2 do not support Th1 cell activation, the effect of the secretions-induced decreased expression of B7.2 and HLA-DR on these cells is not clear. Together, maggot secretions may effect macrophage T cell interactions and this will be the subject of further studies.

Another remarkable finding of this study pertains to the differential effects of LPS on MØ-1 and MØ-2. LPS stimulates a pro-inflammatory responses in MØ-1 and subsequently down-regulates the expression of TLR2 and TLR4 on these macrophages, which is reported earlier as LPS tolerance [33]. These tolerized MØ-1 poorly respond to another challenge with LPS, thus reducing the pro-inflammatory response and preventing excessive reactions against infection and subsequent detrimental effects on the surrounding tissue. In contrast, MØ-2 exert anti-inflammatory and pro-angiogenic activities. However, once the infection is cleared these cells may have to initiate a swift response against a starting/recurring infection. Therefore, the increased expression of TLRs on these cells may act as a positive regulator of inflammation. In agreement, we found that MØ-2 produce relatively high levels of pro-inflammatory cytokines upon stimulation with low, physiological amounts of LPS (0.01 or 0.1 ng of LPS/ml) as compared to high levels of LPS (100 ng/ml). In addition, MØ-2 produce high levels of chemokines upon LPS stimulation indicating that these cells can attract many additional immune cells. Collectively, it would be interesting to further investigate the differences between these two subsets of macrophages and their role in acute and chronic wounds.

What could be the relevance of the present findings? In a normal wound healing process, resident cells like macrophages efficiently detect microbial structures and respond to this by recruiting neutrophils and monocytes to fight off the invading pathogens. Initially, monocytes may differentiate to pro-inflammatory macrophages that regulate the inflammatory process. When the infection recedes due to removal of pathogens and cellular debris, the composition of the local environment will change facilitating differentiation of monocytes to anti-inflammatory/pro-angiogenic macrophages. These cells suppress inflammatory responses directly [19], [34], [35] and indirectly by inducing regulatory T cells [36] and mediate neovascularisation, cell proliferation [20] and subsequent matrix synthesis [21], [22] resulting in repair of the wound. In agreement, we found MØ-1 to produce considerable levels of pro-inflammatory cytokines (e.g. TNF-α, IL-12p40, MIF) whereas MØ-2 produced high levels of IL-10, bFGF and VEGF. Although pro-inflammatory cytokines are essential for acute inflammatory responses, they can be detrimental in chronic wounds were inflammation persists. Histological data exists showing that parts of chronic wounds seem to be stuck in different phases of healing, with loss of synchronicity, which is essential for a rapid healing [37]. Some parts of the wound that are ready for fibroblast proliferation and epidermal resurfacing could be damaged by the inflammatory phase still present in other parts of the wound [38]. Chronic leg ulcers are associated with elevated expression of pro-inflammatory cytokines, like TNF-α and MIF, compared to acute wounds [39]–[41]. These cytokines promote the production of more pro-inflammatory cytokines [42], [43], up-regulate the synthesis of matrix metalloproteinases and serine proteases [24], [42], [44] and activate the reactive oxygen generating system [45], [46]. Together, these pro-inflammatory actions result in extracellular matrix destruction [47]–[49] and inactivation of growth factors and protease inhibitors [44], [50]–[52]. Our results showed inhibited production of pro-inflammatory cytokines by macrophages differentiated in the presence of secretions. These actions of maggots may provide protection against progression towards ongoing inflammation and tissue destruction by these cells in chronic wounds and may result in an environment beneficial for healing. Simultaneously, the increased pro-angiogenic activity of anti-inflammatory macrophages may induce neovascularisation and the concurrent formation of granulation tissue. In agreement, others reported that maggots increase the expression of bFGF in ulcer tissue [53] and induce the formation of granulation tissue [9], [54]. Taken together, the actions of secretions described in this study contribute to the exiting beneficial effects of maggots in diabetic foot ulcers and other chronic wounds unresponsive to conventional therapies.
